# Emergence of Collective Territorial Defense in Bacterial Communities: Horizontal Gene Transfer Can Stabilize Microbiomes

**DOI:** 10.1371/journal.pone.0095511

**Published:** 2014-04-22

**Authors:** János Juhász, Attila Kertész-Farkas, Dóra Szabó, Sándor Pongor

**Affiliations:** 1 Faculty of Information Technology and Bionics, Pázmány Péter Catholic University, Budapest, Hungary; 2 Group of Protein Structure and Bioinformatics, International Centre for Genetic Engineering and Biotechnology, Trieste, Italy; 3 Institute of Medical Microbiology, Semmelweis University, Budapest, Hungary; Semmelweis University, Hungary

## Abstract

Multispecies bacterial communities such as the microbiota of the gastrointestinal tract can be remarkably stable and resilient even though they consist of cells and species that compete for resources and also produce a large number of antimicrobial agents. Computational modeling suggests that horizontal transfer of resistance genes may greatly contribute to the formation of stable and diverse communities capable of protecting themselves with a battery of antimicrobial agents while preserving a varied metabolic repertoire of the constituent species. In other words horizontal transfer of resistance genes makes a community compatible in terms of exoproducts and capable to maintain a varied and mature metagenome. The same property may allow microbiota to protect a host organism, or if used as a microbial therapy, to purge pathogens and restore a protective environment.

## Introduction

Multispecies microbial communities are a major form of life that can coexist with many other organisms. It is well known that the human body carries 10 times more microbial cells than the number of its own cells. One of the many intriguing properties of microbial communities is that they can provide protection to their host organism against infection or colonization by pathogens. Examples include the protective effects of healthy gut microbiota [1], [2], probiotics [3], or the ability of a healthy rhizosphere to fend-off plant pathogenic soil bacteria [4]. Little is known about the mechanisms of such a territorial defense, which is especially intriguing since it emerges in a wide variety of contexts. One of our goals is to understand if horizontal gene transfer (HGT) can contribute to the emergence of protective properties in microbial communities such as the human microbiota.

Horizontal gene transfer (HGT), i.e. the process by which bacteria acquire genetic material from neighboring cells [5] is now considered the key to many important processes, such as, for instance, the spreading of bacterial antibiotic resistance [6]–[8]. Dense microbial communities, such as the human gut microbiota are now considered a hot spot of microbial gene transfer [9]. This is all the more interesting since it was recently discovered that the rate of HGT is apparently eight to nine orders of magnitude faster than previously thought [10]. As a result, rapid microbial evolution is now believed to be a major factor that can shape the community structure of microbial consortia [11]–[13]. The spreading of resistance genes is especially intriguing in this respect, since members of a stable microbial community must be resistant/tolerant to a great number of exoproducts that might be released by the hundreds or thousands of species constituting a consortium. There is no doubt that the natural tolerance of bacteria towards various classes of chemicals may provide a shield against many antimicrobial agents. However, the spread of specific resistance genes is also a plausible mechanism that can explain the formation of mutual resistance within multispecies consortia. We hypothesize that the resistance of coexisting bacteria towards the exoproducts present in a consortium is an important prerequisite for a stable community. One of our goals therefore is to follow the build-up of this property via a computer simulation of HGT between coexisting species.

Competition between coexisting species is often classified into two broad categories: exploitative competition and interference competition [14]–[16]. Exploitative or scrambling competition refers to species competing for exhaustible resources such as nutrients or space. In contrast, interference competition is a process by which competing species try to limit growth of competitors via factors such as antimicrobial substances. A growing body of evidence from experimental and computer simulation studies indicate that resource utilization by different bacterial species can be both cooperative and competitive, and in particular that cheaters can abuse cooperating species [17]. Recently it has been suggested that such a competition between bacteria leads either to a collapse of the community (competitive exclusion), or to a stable coexistence [18], [19], and that the latter can be mediated by a sharing of signals and public goods [20]. Genetic and mechanistic studies of such interference competition in bacteria are relatively recent [21], even though there are many well known classes of antimicrobial compounds that bacteria deploy against other species [22]. The work we report here is concerned with this second class of competition, interference competition. We are particularly interested in how species coexisting in populated niches become accustomed to the interference competition of the other species, and how this acclimation process, that we term community maturation, renders a microbial community resistant against newcomers.

Here we show that simple computational agents capable of taking over resistance genes from their immediate neighbors can form stable and diverse communities that both produce and are resistant to a large number of antimicrobial agents. This complex antimicrobial profile leads to a community capable of keeping invading species away. At the same time, the model also explains why a transplantation of a mixed microbial community into an environment dominated by a single highly resistant species can restore a stable and more mixed equilibrium.

## Results

### The model

**Figure 1:**
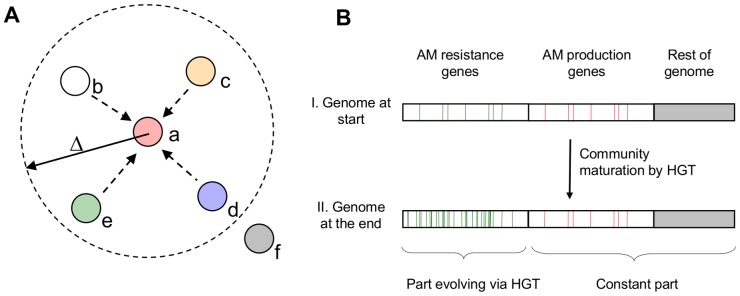
A contact-based model of horizontal transfer in bacteria. A) Agent *A* is in contact with those bacteria that are within its neighborhood (denoted as a dashed circle of radius *Δ*). If bacteria *B–E* have more antimicrobial agent (AM) production genes than the tolerance threshold, *A* will die. If not, *A* will randomly take a number of resistance genes from the cells within this neighborhood. In each simulation step, this calculation is repeated for all agent models within the community and then the agents move randomly (see methods) B). After a number of simulation steps, the agents take up all resistance genes present in the community (green lines), the AM production genes (red lines) and the rest of the genome (grey) remains unchanged.

Briefly, the formation of a multispecies microbial community was simulated with randomly moving computational agents (representing cells) that could randomly acquire ‘genes’ from the neighboring agents they were in contact with, i.e. that were within a certain distance (Figure 1). The computational agents were equipped with randomly generated genomes consisting of two types of genes. The first type encoded the production of an antimicrobial agent (AM, or briefly an antimicrobial, see glossary, Table 1) that could kill susceptible bacteria. The other type of gene encoded specific resistance against one specific AM. The rest of the genome - including the genes responsible for the metabolic repertoire – was not explicitly represented. In addition the computational agents were naturally tolerant to a predefined number of AMs termed the survival threshold (see glossary).

**Table 1:** Glossary of terms.

Glossary of terms
**Antimicrobial agents** or **antimicrobials**. A general term for the drugs, chemicals, or other substances that either kill or slow the growth of microbial cells. Here, we use this term for the substances naturally produced by bacteria.
**Community strength**. We use this term to denote the number of antimicrobials (or resistance genes) produced by a bacterial community.
**Diversity**: Species diversity is the effective number of different species that are represented in a collection of individuals (a dataset).
**Fitness**: The extent to which an organism is able to produce offspring in a particular environment. Here, we use the growth rate of a species as a measure of fitness.
**Horizontal Gene transfer (HGT)** The term refers to the transfer of genes between organisms in a manner other than traditional reproduction. Also termed lateral gene transfer (LGT).
**Invasion, invasivity, invadability**: Invasion is the expansion of a species into an area not previously occupied by it. Here, we use this term for a species taking over a bacterial community. Such a species is termed invasive, and the community is termed invadable.
**Multidrug resistance**: A term used for bacteria resistant to antibacterial agents having different mechanisms of action.
**Natural tolerance**: We use this term to denote the inherent ability of bacteria to grow in the presence of a certain number of antimicrobials in their environment.
**Specific resistance**: The ability of bacteria to grow in the presence of some chemical agents that have a given mechanism of action.

The agents moved randomly on a circular 2D surface and acquired specific resistance genes from their immediate neighbors via horizontal gene transfer. Importantly, only specific resistance genes were exchanged during the simulation. The transfer of genes necessary for the production of an AM, or genes to confer inherent resistance, were set to take much longer than any modeling experiment we intended to run. As a result, the pool of resistance genes became homogeneous over the experimental period i.e. all species contained the same resistance genes. The other parts of the genome remained constant with each species producing a different set of AMs, and a different metabolic repertoire.

For community evolution experiments, a given number (usually 50) of “naïve” or starting agents were created first. The naïve agents carried an equal number (typically 1 to 50) of randomly chosen AM production genes as well as the matching specific resistance genes. As a result, all computational agents had different genomes i.e. they represented different species. Before simulation started, the computational agents were randomly placed in a 2D circular area, and then they were allowed to complete their life cycles that included random movement, horizontal gene transfer, division and/or extinction. As the computational agents were also allowed to divide during the simulations, certain species became more abundant while others disappeared. The simulation was left to proceed until no more HGT occurred in the community (see Methods for more details).

### Simulation outcomes: Community genotypes

**Figure 2:**
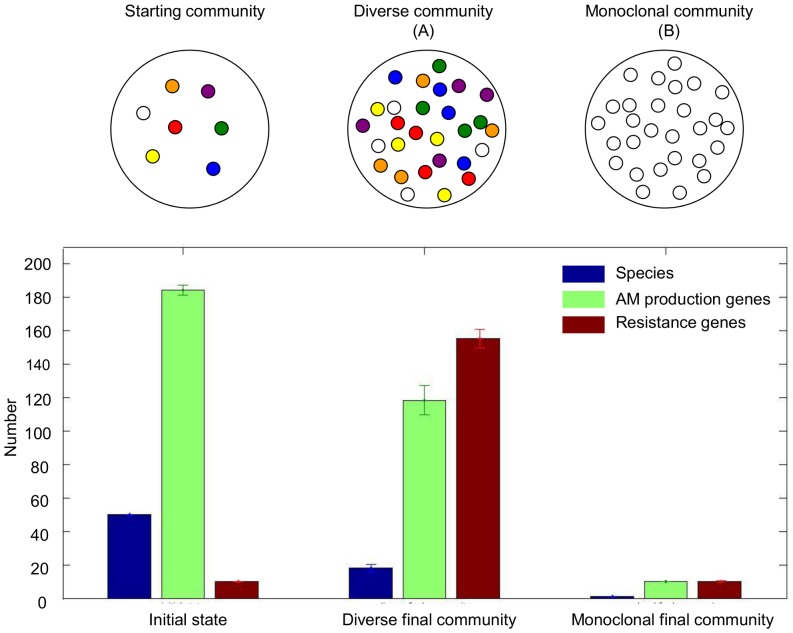
Composition of the model communities. If horizontal gene transfer is possible, the starting community of computational agents (left) can evolve to a diverse community (A) in which substantial parts of the starting species are preserved. If horizontal gene transfer is not effective, a non-diverse, “monoclonal” community will form, with essentially one species (B). The numbers in the diagram represent the average, the error bars the standard deviation, respectively, calculated from 100 simulations.

During the simulations we monitored the number of species present, the number of AMs produced, as well as the genome of all computational agents. It became readily apparent that the formation of a diverse community was primarily determined by a few parameters: the speed of horizontal gene transfer, the level of nonspecific resistance as well as the number of contacts made by the agents which in turn was determined by the density of the agent population and/or the speed of random movement. Mapping out the parameter space then consisted of carrying out simulation experiments by varying agent parameters in a grid-like fashion. This exercise required a large number of simulations, each of them resulting in a final distribution of species and genomes to be described in numerical as well as biological terms. In order to facilitate evaluation, we carried out preliminary experiments in order to explore the types of simulation outcomes. Interestingly, only two outcomes were observed (Figure 2):

A) Diverse community (A in Figure 2). In this case, the agents form a homogeneous, mixed population typically consisting of about 20 species that produce about 100 AMs. One particular species produces only a few AMs but is resistant to all AMs produced within the community (II. in Figure 1B). This outcome is analogous to the formation of a healthy and stable microbial community with a community metagenome that contains a large number of AM genes, and a matching number of resistance genes. Given the relatively large number of AMs produced, and the large number of resistance genes in each constituent species, such a community is unlikely to be easily invaded by external species.

B) No diversity (close to monoclonal community, B in Figure 2). Only one of the species survives, the others die out. An analogous phenomenon is well known in microbiology laboratories: *ad hoc* mixtures of bacterial species generally do not remain unchanged for long, usually one or few species survive [23], [24]. In the simulations, this scenario is observed if the given conditions do not allow HGT to take place, meaning that the only surviving species will have one of the starting genomes.

**Figure 3:**
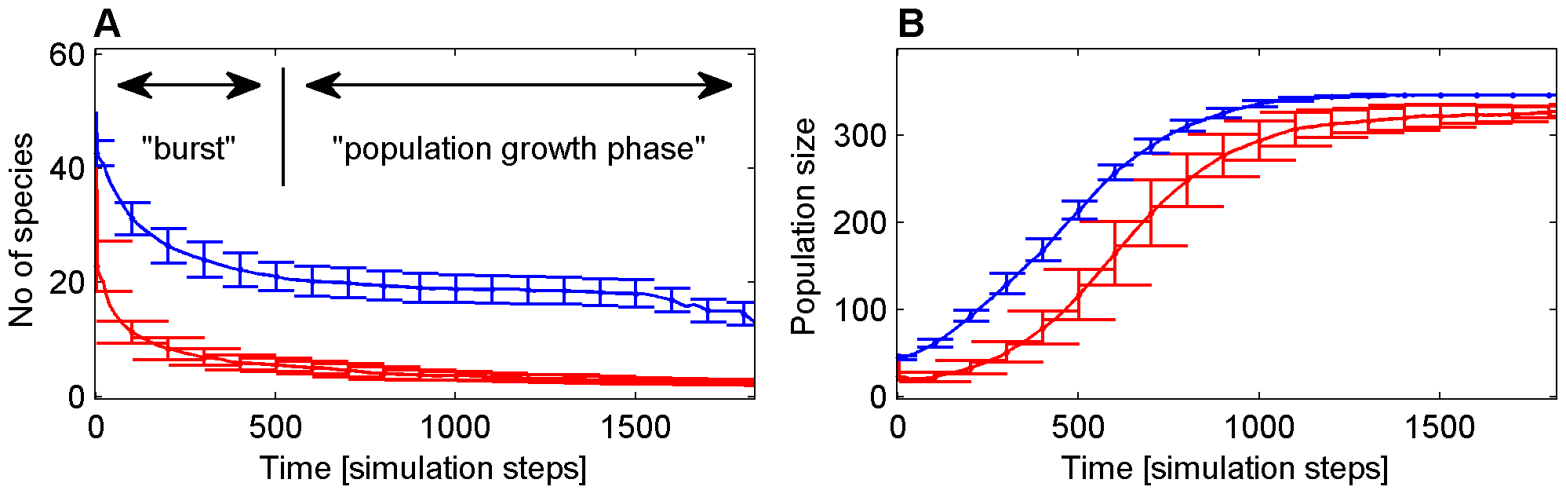
Community evolution in time, measured in simulation steps (arbitrary units). A) number of species and B) total number of computational agents as a function of time. Blue (outcome A): Diverse community; red (outcome B): monoclonal community. The error bars represent standard deviation of the mean in 100 experiments.

The evolution process seemed to follow the same general course both for the diverse and for the monoclonal outcomes (Figure 3). It consisted of two clearly distinguishable phases: 1) An initial burst phase, in which the number of surviving species drops to a lower level, and 2) a subsequent growth phase in which the number of agents increases but the number of species remains approximately constant.

### Factors governing community evolution

**Figure 4:**
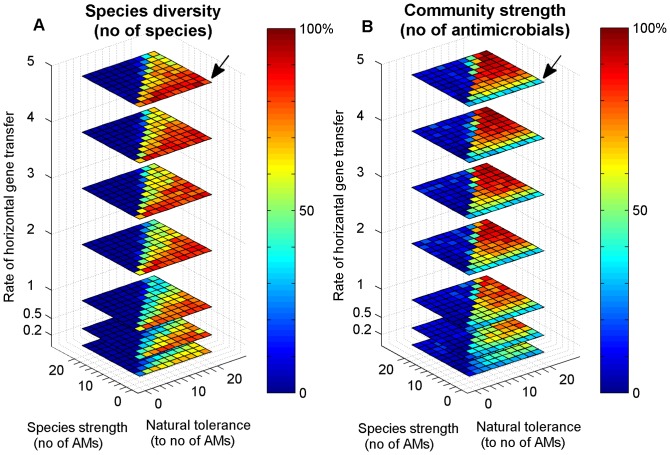
Strength and diversity of the community depends on the rate of horizontal gene transfer and on the properties of the individual species (number of antimicrobials produced and the natural tolerance to antimicrobials). Note that HGT promotes community strength (top planes vs. bottom planes). Species carrying few AM genes can form a diverse, but weaker community (black arrows).

**Figure 5:**
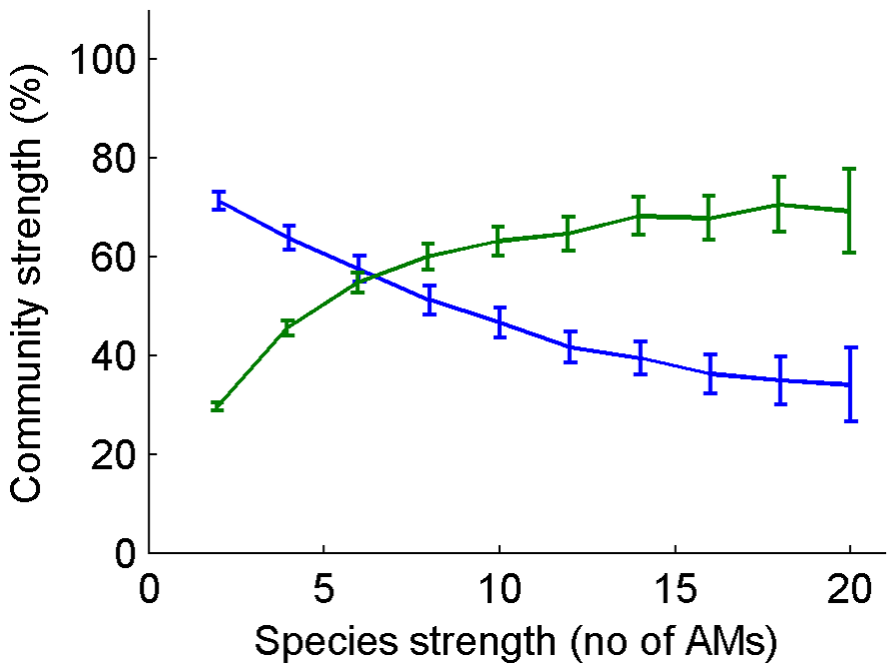
Community strength and community diversity (Y-axis) are adversely affected by the strength of the individual species (X-axis). Note that this figure is a collapsed version of Figure 4.

In order to find out the conditions that favor the formation of diverse and strong communities that produce a large number of antimicrobials, we carried out a large number of simulation experiments by systematically varying parameters such as the rate of horizontal gene transfer, the tolerance threshold (innate, non-specific tolerance) and the strength of the individual species (number of AM/resistance gene-pairs) (Figures 4–5). In Figure 4, the blue areas indicate those conditions where communities are unable to form (which corresponds to the monoclonal population in Figure 2). This outcome is seen either when there is no HGT (the rate is zero or very slow), or the species produce more antimicrobials than they can tolerate on average (this results in a diagonal division). On the other hand, the results in Figure 4 also show that communities can form under a large variety of parameter combinations. Community strength (expressed as the number of AM producing and resistance genes) depends on the rate of HGT, i.e. strong communities that produce many AMs form if HGT is intensive during the maturation process. This can be seen in the left panel of Figure 4 as an increase of the red area towards higher HGT values. The effect of species strength is summarized in Figure 5. It is apparent that strong species form less diverse communities, even though the community will form a large number of AMs. Conversely, weak species that produce a small number of AMs can form communities more easily, but the resulting community will be weak in terms of AMs produced. We note that the genes representing the metabolic capabilities of the individual species are not explicitly represented in this model, i.e. species diversity is a direct measure of a community's metabolic repertoire. As a result, the optimum lies at intermediate strength values, i.e. metabolically diverse communities that contain a relatively large number of AMs can be formed by species of intermediate strength. In simple terms, superbugs are not team players in this model.

We also carried out the simulations with various other parameter settings and found that diversity does not emerge in conditions that allow exceedingly high levels of agent/agent contact. Such conditions included high population densities or high rate of agent movements (data not shown).

### Competition experiments

**Figure 6:**
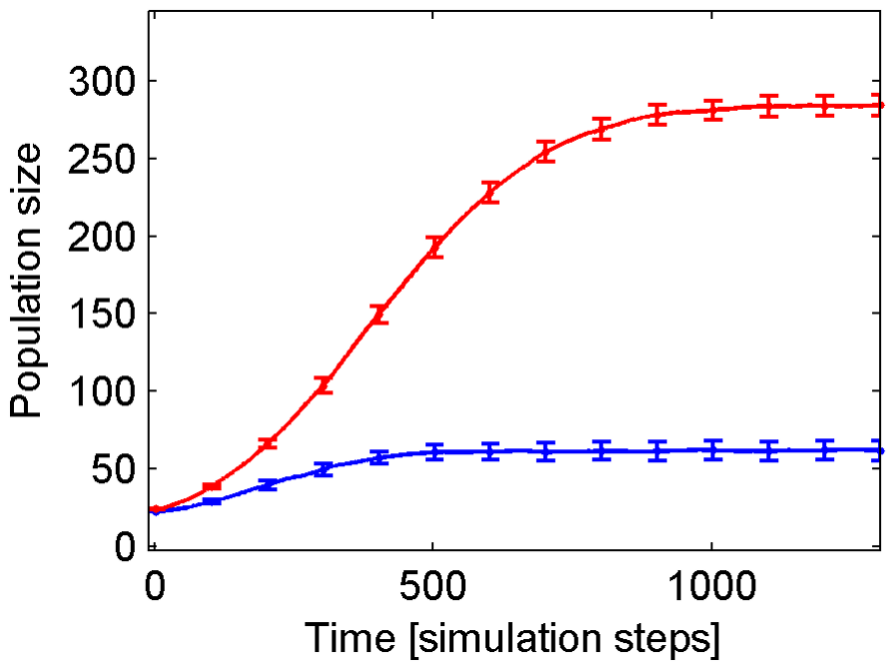
Competition of communities differing in their nonspecific resistance to antimicrobial components. The natural tolerance (nonspecific resistance) threshold was 10 for the blue and 15 for the red community, respectively. The numbers represent an average of 25 simulations; the error bars represent standard deviation of the mean. The starting communities consisted of 200 cell agents from each of the populations.

The above tendencies can be validated by population competition experiments. As an example, Figure 6 shows the competition of two populations that differ only in their natural tolerance property. It can be seen that the red population that can tolerate more AMs grows faster and transmits more species to the final community than the blue population that is nonspecifically less resistant. It is worth noting that once a population of blue and red agents formed, it remained constant for the rest of the simulation. In other words, once the two species tolerate the AMs of each other, the community does not necessarily change, at least according to the present HGT model.

### Territorial defense: protection against invading bacteria

Invasion was modeled with naïve or mature communities as described in the Methods. To a community of 50 computational agents we added 25 “invader” agents of a single species in which the species strength, i.e. the number of AM producing and resistance genes, varied from experiment to experiment (Table 2). We found that a naïve community could be easily taken over by the invader population, but the invaders were virtually eliminated by a mature community. Only highly resistant “superbugs” (rows 4–7 in Table 2) could grow in a mature community. The results show that a mature community is likely to be more resistant to external attack than a naïve community in which the constituent species are not acclimatized to the AMs of each other.

**Table 2:** Territorial defense: vulnerability of naïve and mature communities to an invading species producing varying number of antimicrobial agents.

	Community 1 (invader)^3^				Community 2: naive (resident)^1^^,^ 3	Community 2: mature (resident)^2^^,^ 3
	#cells	#species	#AMs	#resistances	% of survivors^3^:	% of survivors^3^:
1	25	1	10	10	25	100
2	25	1	20	20	0	100
3	25	1	50	50	0	100
4	25	1	75	75	0	90
5	25	1	100	100	0	53
6	25	1	125	125	0	17
7	25	1	150	150	0	3

1 Naive community: 50 cells, on average corresponding to 50 species, harboring a total of 181 AM production genes, and the same number of resistance genes.

2 Mature community: 50 cells, corresponding to 15 species harboring a total of 109 AM genes and 180 resistance genes on the average.

3 All numbers indicate the average of 100 experiments.

### Microbial therapy: Purging a pathogen with a healthy microbial community

We also considered an opposing scenario whereby a highly resistant resident population of a single species might be present in a particular environmental niche and whether a multi-species ‘invading’ consortium could overcome it. This is a biologically plausible scenario in that clearing antibiotic resistant pathogens from host organisms is a recurrent and serious problem in both medicine and agriculture. To test this we investigated a multi-resistant pathogen capable of forming spores – analogous to the medically important *Clostridium difficile*. Broad-spectrum antibiotics are often used in health care settings following surgery or infection and can easily wipe out the resident microbiota of the gastrointestinal tract and/or other physiological regions. This may (or may not) also include the vegetative cells of *C. difficile* if present. However the spores of such species will survive. Upon cessation of the antibiotic treatment, the microbiota starts to grow again; however this time with *C. difficile* at proportionally different levels (partly because of the spores and partly because it may be less impacted by the antibiotic than the other microbes). Our model predicts that once a community forms, its composition will not change by itself. Accordingly, a distorted community will not necessarily revert to its original ‘healthy’ state upon cessation of antibiotic treatment – an effect that has been widely documented via experimental and clinical investigations [25]. Treating a dysbiotic microbiota with healthy microbiota seems a promising avenue and maybe relevant in a wide variety of treatment targets other than recurrent *C.difficile* infections [26]–[29].

In order to simulate this situation, we constructed a series of resident pathogens that contained a varying number of AM production and resistance genes, and treated it with a mature community of cell agents, that on the average contained less genes than the pathogen, but where the community produced a large number of AMs. The results in Table 3 show that the pathogen could in fact be purged under certain conditions, unless it contained an unusually high number of resistance genes (rows 5-6 in Table 3).

**Table 3:** Microbial therapy: Purging a pathogen with a transplant of a mature microbial community.

	Community 1 (transplant)^1^				Community 2: single pathogen species (resident)^1^^,^ 2			
	#cells	#species	#AMs	#resistances	#cells	#AMs	#resistances	% survivors
1	25	10	81	180	25	20	20	0
2	25	10	81	180	25	20	50	0
3	25	10	81	180	25	20	75	0
4	25	10	81	180	25	20	100	0
5	25	10	81	180	25	20	125	2
6	25	10	81	180	25	20	150	27

1 Mature community.

2 All numbers indicate the average of 100 experiments.

## Discussion

We have presented a contact-based computational model of HGT in which random moving computational agents can acquire resistance genes from their neighbors. The simulations suggest that HGT can in fact facilitate the formation of resistant communities that protect themselves by means of antimicrobial components. Such mature communities are apparently difficult to invade by randomly arriving pathogens (Table 2) – the collective territorial defense can protect both the community and subsequently any host organism. In addition, an implant of microbiota may be capable of effectively purging a pathogen from an organism or environment niche (Table 3). Taken together, our model predicts that a mature microbial community has properties analogous to multi-target drugs or cocktail therapies against which it is difficult to raise effective resistance [30]. In other words we believe that the presence of a large number of antimicrobial agents may be crucial for the collective defense and stability of natural microbial communities by keeping invaders away. This property may be useful in designing bacterial communities for therapeutic or probiotic purposes. Another implicit suggestion of the model is that HGT helps a community preserve a varied metabolic repertoire and makes it compatible in terms of exoproducts as well as capable to maintain a varied metagenome.

In terms of limitations of this modeling approach, superbugs (i.e. computational model agents equipped with a large number of AM production and/or resistance genes) can always invade a mature community. However we need to point out that the model does not indicate how resistant a superbug needs to be to actually achieve this. That is to say, the model works with symbolic parameters only and so this result should be interpreted only as a general indication of superbugs being dangerous (which is a realistic prediction), and not as saying that such superbugs do or can exist.

Another limitation of the model is that it does not contain a metabolic component; it only deals with the compatibility of AM production and resistance within a community, and not with resource competition. In the context of community evolution our model points to the plausible fact that members of a stable bacterial consortium must become compatible in terms of secondary metabolites and predicts that HGT may be one of several mechanisms that mediate this process. On the other hand, compatibility does not mean stability. Stability of the community will be determined by resource competition between the compatible species. In other words, we believe that a structure of a community will depend both on interference competition and on resource competition.

In summary, we argue, based on computer simulations, that the resistance of a community against invaders, and the capability of a community to purge a pathogen from an environmental niche, can be explained by bacteria exchanging resistance genes and thereby maturing into a community that has a variety of metabolic and antimicrobial producing competences. Based on these findings, we suggest that HGT may be a viable target to study experimentally with respect to the stability of multi-species microbial communities and their resistance characteristics. Such investigations are likely to be highly relevant in terms of the development of treatment approaches for intractable infections that are currently (or are likely to be in the near future) resistant to multiple anti-microbial therapies.

## Methods

The model consists of agents (representing cells) that are dimensionless points randomly moving on a 2D surface. The simulation proceeds in discrete time-steps, and at every time point the agents update their genomes as described below in detail. The model works with symbolic parameters, summarized in Table 4a-3c.

**Table 4:** Model parameters for initialization

Parameters	Description	Default value
Number of bacteria	Number of agents at the beginning randomly generated	50
*Dish radius*	Radius of the circle in which the cell agents can move	2
Number of AM	Number of antimicrobial factors	200
Number of resistances	Number of resistance genes	200
No of genes	Number of randomly chosen AM-resistance gene pairs in a cell-agent at the beginning of the simulation	10
Termination	The condition when the simulation stops	95 % of the occuring resistance genes were spread
Model parameters for horizontal gene transfer (HGT) and movement		
**Parameters**	**Description**	**Default value**
ST	Survival threshold: The number of non-specific resistance that an agent can endure	4
Δ	The radius within which a specific resistance gene takes effect	0.2
HGT frequency	The frequency of HGT per iteration for one agent	1
*d*	The length of a step to a random direction	0.05
Model parameters for population related variables		
**Parameters**	**Description**	**Default value**
*N_max_*	Maximum size of the population	50
*µ*	Division rate, the percentage of the population that divides	0.1
Division frequency		After every 10 iterations
Death rate	The percentage of the population that dies	0.1
Death frequency		After every 40 iterations

### Space and movement

The agents move within a unit circle on a 2D surface (similar to a Petri dish). At the beginning of the simulation, agents are randomly placed within the unit circle, with the location of agent *A* at time *t* denoted by 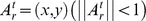 At each time step, the agents move a step of length *d* in a random direction αthat can be formally denoted as: 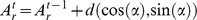 (equation 1). When an agent moves outside the unit circle, it is relocated back within the circle by 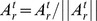.

### Cell-agents

200 antimicrobial factor producing genes (AMP) and 200 antimicrobial resistance genes (AMR) are denoted by numbers 1,…,200. Agents have a random genome, and a randomly chosen subset of AMP and AMR, respectively. Formally, a predetermined number of antimicrobial factor producing genes selected from 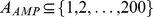 are assigned to an agent A, and the matching resistance genes selected from 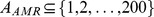 are also assigned to it.

It is assumed that an antimicrobial (AM) acts within a given radius Δ_AM_ around agent *A* that produces it. We call the circle of radius Δ the “contact neighborhood” of agent *A* and denote the set of agents within this circle by 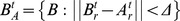. Agents in *B_A_^t^* produce a number of AMs, against which agent *A* may or may not be specifically resistant. NAM is the number of AMs against which agent *A* does not have a specific resistance gene. Agent *A* dies (i.e. it is deleted from the simulation) if NAM is greater than the survival threshold *ST*. Formally, an agent A dies if 
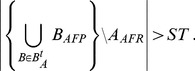
(equation\;2)


*ST* is a measure of aspecific resistance since ST is the number of antimicrobial factors that an agent can tolerate.

Agents are capable of acquiring new resistance genes from other agents within the contact neighborhood via HGT. This is formally written as 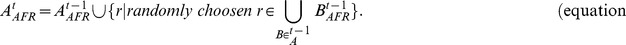
(equation\;3)


The genes acquired via HGT are randomly picked from the genes present in the neighboring cells, and if an agent randomly picks a gene it already has, no transfer will occur. This ensures that the number of HGT slows down over time.

Population growth and decay

**Figure 7:**
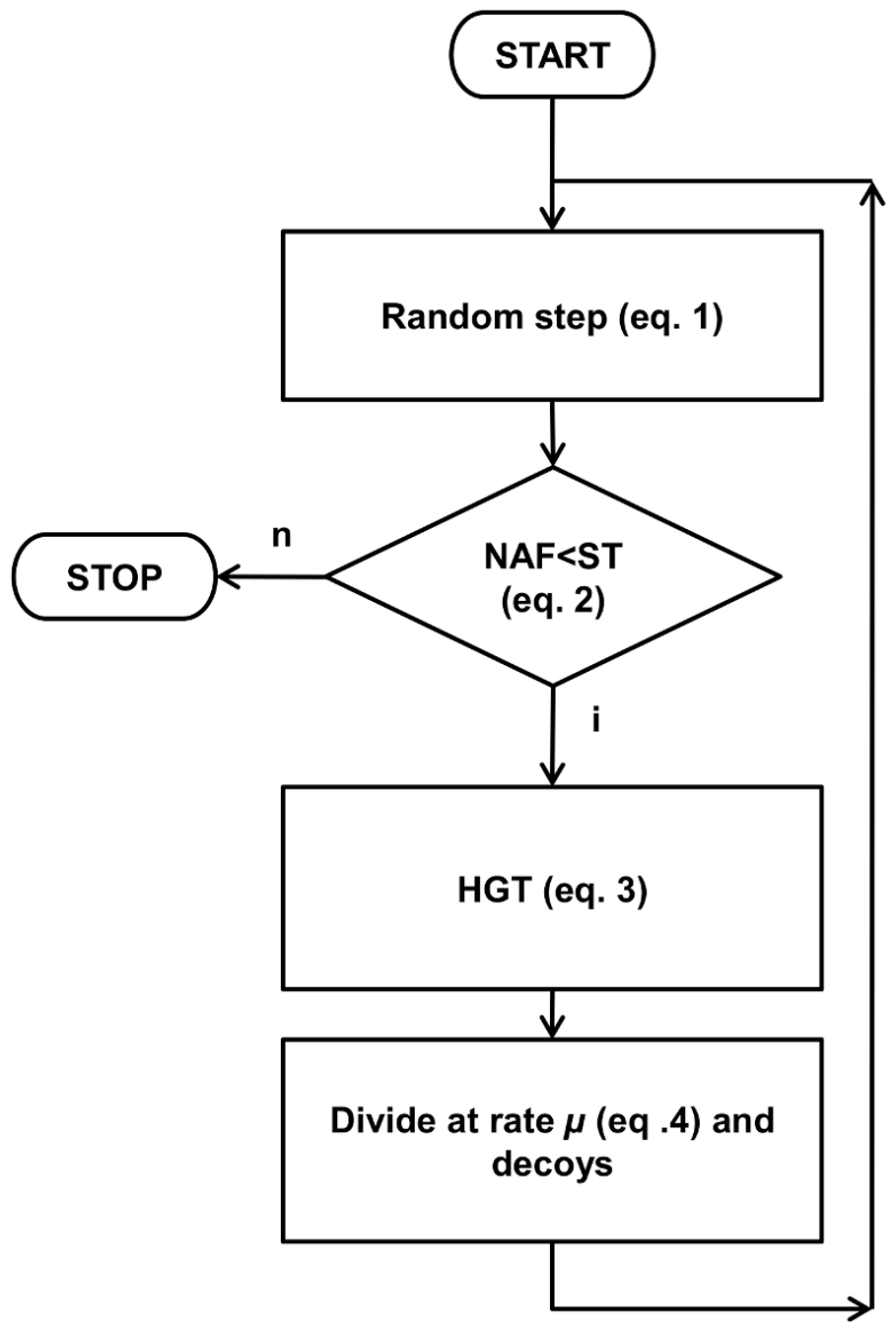
Flowchart representation of a cycle of an agent. Note that the division rate *µ* depends on the size of the population according to equation 4.

At given time intervals, a certain percentage of the agents divide i.e. produces an offspring that has the same genome as the parent. The agent population growth follows a sigmoid curve described by the Richards model [31]. The portion of the agents to be duplicated is calculated by
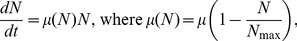
(equation\;4) where *µ* is the maximum specific growth rate, *N* is the size of the population at time *t*, and *N_max_* is the maximum population that can be sustained (set to 400 in all of our experiments). At given intervals a certain percentage of the population dies (i.e. some randomly selected agents are deleted). The complete life cycle of an agent can be seen in Figure 7.

### Community evolution experiments

At the beginning of the experiment, a starting population of 400 agents was created by randomly choosing a specified set of AM production and resistance genes. Theses agents, which we term as *naïve*, were randomly placed on the circular surface, according to an even distribution. During the simulation, the agents performed the functions described in Figure 7 and equations 1-3, at every given time step. As a resulţ the specific resistance genes spread in the population, and the population grew according to the sigmoid curve described by equation 4. The simulations were terminated when the change in the population content or the genome was not significant compared to the previous iterations. More precisely, the simulations were terminated when either no agents had died according to equation 2 in the previous 50 iterations or when 95 per cent of the resistance genes had been spread via HGT. We term the resulting agent populations as *mature.*

Each agent in the starting population was given a cell line identifier, which was also passed on to its progeny. The starting and finishing agent populations were characterized by the number of cell lines and number of AMP and AMR genes present in the population. Simulations were run in 10 repetitions, and results were presented as progress curves, representing the average of a given variable with error bars representing the standard deviation of the mean.

### Population competition experiments

For the competition experiments, we used mature populations produced by the evolution experiments described in the previous section. As a rule, two populations of equal size were placed on the circular surface and the simulations were allowed to proceed as described above. The simulations proceeded until the populations either stabilized or one of them died out.
